# Epidemiological associations between hyperuricemia and cardiometabolic risk factors: a comprehensive study from Chinese community

**DOI:** 10.1186/s12872-015-0116-z

**Published:** 2015-10-16

**Authors:** Shihui Fu, Leiming Luo, Ping Ye, Wenkai Xiao

**Affiliations:** Department of Geriatric Cardiology, Chinese People’s Liberation Army General Hospital, Beijing, 100853 China; Department of Cardiology and Hainan Branch, Chinese People’s Liberation Army General Hospital, Beijing, 100853 China

**Keywords:** Hyperuricemia, Cardiometabolic risk factor

## Abstract

**Background:**

This study aimed to assess the associations of serum uric acid (SUA) levels and hyperuricemia with cardiometabolic risk factors in a Chinese community-dwelling population.

**Methods:**

A large cohort of 4706 residents was enrolled in this study. Physical examinations and laboratory tests were performed following a standardized protocol. Multiple linear and logistic regression analyses were conducted with adjustment of cardiometabolic risk factors including age, sex, body mass index (BMI), blood pressure (BP), triglyceride (TG), high-density lipoprotein-cholesterol (HDL-c), low-density lipoprotein-cholesterol (LDL-c) and fasting blood glucose (FBG) levels using SPSS version 17 software.

**Results:**

The prevalence of hyperuricemia was 7.6 %. There were significant differences in age, BMI, BP, TG, HDL-c, LDL-c and FBG levels and the proportion of men between participants with and without hyperuricemia. Multiple linear regression analysis showed that SUA levels were positively associated with age, sex, BMI, BP, TG and LDL-c levels, but negatively associated with HDL-c and FBG levels. Multiple logistic regression analysis showed that per unit increase in age was associated with a 1.014 times higher odds of the presence of hyperuricemia. Men had a 1.858 times higher odds of the presence of hyperuricemia compared with women. Per unit increases in BMI, BP, TG and LDL-c levels were associated with 1.103, 1.016, 1.173 and 1.200 times higher odds of the presence of hyperuricemia, respectively. Per unit increases in HDL-c and FBG levels were associated with 0.616 and 0.900 times lower odds of the presence of hyperuricemia, respectively.

**Conclusions:**

In a Chinese community-dwelling population, age, sex, BMI, BP, TG, HDL-c, LDL-c and FBG levels are cardiometabolic risk factors that are significantly associated with SUA levels, as well as the presence of hyperuricemia.

## Background

Serum uric acid (SUA) is an end product of purine catabolism in humans and higher primates. In parallel with rapid development of cardiometabolic risk factors, including aging, obesity, high blood pressure (BP), lipids and glucose, the prevalence of hyperuricemia is also rising in developing and developed countries [1, 2]. Elevated levels of SUA are associated with increased cardiovascular morbidity and mortality in subjects who already have cardiometabolic risk factors [3–5]. The role of SUA in elevation of cardiovascular morbidity and mortality remains unclear. Further studies on cardiometabolic risk factors associated with SUA levels might uncover previously unrecognized pathophysiological mechanisms between SUA levels and cardiovascular morbidity and mortality [6]. Recently, SUA levels were reported to be related to cardiometabolic risk factors including advanced age, obesity, high blood pressure, lipids and glucose [7, 8]. However, the specific associations between SUA levels and cardiometabolic risk factors remain undefined. Moreover, there is evidence that hyperuricemia is not uncommon in China. However, studies in Chinese community-dwelling population on the associations between SUA levels and cardiometabolic risk factors are scarce [6]. Therefore, we conducted a comprehensive study involving a large cohort of Chinese community-dwelling population to assess the potential associations of SUA levels and hyperuricemia with cardiometabolic risk factors,.

## Methods

### Study population

This study enrolled a large cohort of 4706 community-dwelling residents in the Beijing area. Participants were consecutively referred by physicians in the local health service center for a medical examination between May 2007 and July 2009. Recruited individuals were Han in origin and older than 18 years. Community-dwelling residents were defined as residents who have lived in the communities for at least 6 months. A stratified cluster sampling design was used in this survey. In the first stage of sampling, three districts (Fengtai, Shijingshan, and Daxing) were selected from 18 districts in Beijing. In the second stage of sampling, four communities were selected from these districts. In the third stage of sampling, participants were selected from these communities to represent the overall Beijing population. The study protocol was approved by the Ethics Committee of Chinese People’s Liberation Army General Hospital (Beijing, China). Each participant provided written informed consent to be included in the study.

### Physical examinations

Physical examinations were performed following a standardized protocol. Anthropometrics were measured in the clinic, with participants wearing light clothing and no shoes. Height was measured in centimeters using a wall-mounted measuring tape, and weight was measured in kilograms using a digital scale. Body mass index (BMI) was calculated as weight (kg) divided by height squared (m^2^). BP was measured by well-trained personnel on the right arm of the seated participant, after at least 5 min of rest, with a mercury sphygmomanometer (Yuwell Medical Equipment & Supply Co., Ltd., Jiangsu, China) and an appropriately-sized cuff. Two sequential BP measurements were obtained, and the average was used for analysis. Pulse pressure (PP) was the difference between systolic BP and diastolic BP (DBP).

### Biochemical measurements

Venous blood specimens were routinely collected by venipuncture between 8 am and 10 am in overnight fasting subjects after they had been in the supine position for 5 min. After blood was drawn, specimens were routinely stored and delivered to the central laboratory in the Department of Biochemistry, Chinese People’s Liberation Army General Hospital, on the same date. Fasting blood glucose (FBG), triglyceride (TG), high-density lipoprotein-cholesterol (HDL-c), low-density lipoprotein-cholesterol (LDL-c) and SUA levels were measured directly by a qualified technician blinded to clinical data using enzymatic assays (Roche Products Ltd., Basel, Switzerland) on a full automatic biochemical autoanalyzer (COBAS c6000; Roche Products Ltd.). The criteria used to define hyperuricemia were SUV levels greater than 360 μmol/L and 420 μmol/L in women and men, respectively [9].

### Statistical analysis

All continuous variables are shown as mean and standard deviation (SD, normal distribution) or median and interquartile range (non-normal distribution). Categorical variables are shown as number and proportion. Continuous variables were compared using the Student’s *t*-test (normal distribution) or Mann–Whitney *U* test (non-normal distribution). Comparison of categorical variables was conducted by the chi-square test. To evaluate the associations between SUA levels and cardiometabolic risk factors as continuous variables, Pearson’s correlation or Spearman’s correlation analysis was used in univariate analysis, and multiple linear regression analysis (stepwise) was also conducted with adjustment of cardiometabolic risk factors including age, sex, BMI, BP, TG, HDL-c, LDL-c and FBG levels; SUA levels as skewed variable were logarithmically transformed to improve normality prior to this analysis. In addition, to better assess cardiometabolic risk factors associated with hyperuricemia, multiple logistic regression analysis (backward stepwise) was conducted after adjusting for cardiometabolic risk factors including age, sex, BMI, BP, TG, HDL-c, LDL-c and FBG levels. Stepwise regression analyses were based on a statistical significance test criterion (*p* values). All analyses were carried out using SPSS version 17 software (SPSS Inc., Chicago, IL, USA) and the level of statistical significance was always set at *p* < 0.05.

## Results

### General characteristics

For all of the 4706 individuals, the median age was 51 years (range, 18–96 years), and 2283 (48.5 %) were men. The prevalence rate of hyperuricemia, hypertension, diabetes and a history of cardiovascular disease was 7.6 % (359 participants), 39.6 % (1864 participants), 10.7 % (505 participants) and 11.6 % (547 participants), respectively. There were significant differences in age, BMI, PP, DBP, TG, HDL-c, LDL-c and FBG levels and the proportion of men between participants with and without hyperuricemia (all *p* < 0.05; Table 1).

**Table 1:** Characteristics of all individuals divided by the presence of hyperuricemia or not

Characteristics	All participants (*n* = 4706)	Hyperuricemia (*n* = 359)	Control (*n* = 4347)	*P*-value
Age (year)^a^	51 (39–62)	56 (42–69)	51 (39–61)	<0.001
Men (%)^b^	2283 (48.5)	217 (60.4)	2066 (47.5)	<0.001
BMI (kg/m^2^)^a^	25.51 (23.15–28.06)	27.34 (24.98–29.74)	25.35 (23.03–27.92)	<0.001
PP (mmHg)^a^	48 (40–58)	53 (43–64)	48 (40–58)	<0.001
DBP (mmHg)^a^	79 (70–84)	80 (72–86)	78 (70–84)	<0.001
TG (mmol/L)^a^	1.36 (0.95–2.00)	1.93 (1.40–2.91)	1.33 (0.93–1.91)	<0.001
HDL-c (mmol/L)^a^	1.38 (1.17–1.62)	1.26 (1.06–1.48)	1.39 (1.18–1.63)	<0.001
LDL-c (mmol/L)^a^	2.75 (2.27–3.26)	2.96 (2.46–3.43)	2.73 (2.25–3.25)	<0.001
FBG (mmol/L)^a^	4.98 (4.64–5.41)	5.08 (4.68–5.54)	4.97 (4.63–5.39)	0.004

*BMI* body mass index, *PP* pulse pressure, *DBP* diastolic blood pressure, *TG* triglyceride, *HDL-c* high-density lipoprotein-cholesterol, *LDL-c* low-density lipoprotein-cholesterol, *FBG* fasting blood glucose

^a^Median (interquartile range)

^b^number (proportion)

### Cardiometabolic risk factors associated with SUA levels

Simple correlation analysis showed that SUA levels were positively related to age, sex, BMI, PP, DBP, TG, LDL-c and FBG levels, but negatively related to HDL-c levels (all *p* < 0.05; Table 2). Multiple linear regression analysis showed that SUA levels were positively associated with age, sex, BMI, PP, TG, and LDL-c levels, but negatively associated with HDL-c and FBG levels (all *p* < 0.05; Table 2).

**Table 2:** Cardiometabolic risk factors associated with serum uric acid levels in simple correlation and multiple linear regression analyses

Variables	Simple correlation		Multiple linear regression	
	Correlation coefficient	*P*-value	β-value	*P*-value
Age (year)	0.066^a^	<0.001	0.071^a^	<0.001
Men/women	0.477^a^	<0.001	0.458^a^	<0.001
BMI (kg/m^2^)	0.233^a^	<0.001	0.166^a^	<0.001
PP (mmHg)	0.117^a^	<0.001	0.041^a^	0.003
DBP (mmHg)	0.139^a^	<0.001	−0.022	0.087
TG (mmol/L)	0.328^a^	<0.001	0.148^a^	<0.001
HDL-c (mmol/L)	−0.291^b^	<0.001	−0.071^b^	<0.001
LDL-c (mmol/L)	0.092^a^	<0.001	0.072^a^	<0.001
FBG (mmol/L)	0.046^a^	0.002	−0.115^b^	<0.001

*BMI* body mass index, *PP* pulse pressure, *DBP* diastolic blood pressure, *TG* triglyceride, *HDL-c* high-density lipoprotein-cholesterol, *LDL-c* low-density lipoprotein-cholesterol, *FBG* fasting blood glucose

^a^Positive

^b^negative

### Cardiometabolic risk factors associated with hyperuricemia

Multiple logistic regression analysis (Table 3) showed that per unit increase in age was associated with a 1.014 times higher odds of the presence of hyperuricemia (*p* < 0.05). Men had a 1.858 times higher odds of the presence of hyperuricemia compared with women (*p* < 0.05). Per unit increases in BMI, PP, TG and LDL-c levels were associated with 1.103, 1.016, 1.173 and 1.200 times higher odds of the presence of hyperuricemia, respectively (all *p* < 0.05). Per unit increases in HDL-c and FBG levels were associated with 0.616 and 0.900 times lower odds of the presence of hyperuricemia, respectively (all *p* < 0.05).

**Table 3:** Cardiometabolic risk factors associated with hyperuricemia in multiple logistic regression analysis

Variables	OR	95 % CI	*P*-value
Age (year)	1.014^a^	1.005–1.023	0.002
Men/women	1.858^a^	1.464–2.359	<0.001
BMI (kg/m^2^)	1.103^a^	1.069–1.137	<0.001
PP (mmHg)	1.016^a^	1.008–1.024	<0.001
DBP (mmHg)	0.999	0.989–1.010	0.866
TG (mmol/L)	1.173^a^	1.103–1.247	<0.001
HDL-c (mmol/L)	0.616^b^	0.415–0.914	0.016
LDL-c (mmol/L)	1.200^a^	1.036–1.390	0.015
FBG (mmol/L)	0.900^b^	0.819–0.989	0.028

*OR* odd ratio, *CI* confidence interval, *BMI* body mass index, *PP* pulse pressure, *DBP* diastolic blood pressure, *TG* triglyceride, *HDL-c* high-density lipoprotein-cholesterol, *LDL-c* low-density lipoprotein-cholesterol, *FBG* fasting blood glucose

^a^Positive

^b^negative

## Discussion

Previous studies in the United States have shown that, in men, SUA levels increase between 10 and 16 years of age and reach a peak at approximately 20 years, whereas, in women, SUA levels increase at approximately 50 years [10]. In a previous univariate analysis on Chinese people, SUA levels of male participants reached a peak between 13 and 18 years of age and slightly decreased after 18 years, whereas SUA levels of female participants were stable before 18 years and slightly decreased between 19 and 44 years, and then increased in the mid to older age groups (≥45 years) [1]. Another Chinese study showed that, in women, age was highly significantly correlated with SUA levels in univariate and multivariate analyses, whereas, in men, a weak correlation was found only in univariate analysis [6]. The current study showed that there was a positive association between age and SUA levels, and an increase in age was significantly associated with an increased odds of hyperuricemia. In addition, men had a higher odds of hyperuricemia compared with women. Sex differences in SUA levels might be induced by urate-depressing action of estrogens [10].

A report from 8000 men of Japanese ancestry living in Hawaii proposed that weight was the most important correlate with SUA levels [11]. In Melanesians and Asian Indians, BMI was significantly correlated with SUA levels [12]. Another multivariate analysis on Chinese people also confirmed that BMI was an important factor associated with hyperuricemia [1]. Consistent with these studies, our data showed that there was a significant association between BMI and SUA levels, and an increase in BMI was significantly associated with an increased odds of hyperuricemia.

Using univariate and multivariate analyses, a relationship between BP and SUA levels has been demonstrated in several studies [13, 14]. However, in Okada et al’s study, BP was not selected as a variable related to SUA levels in multiple linear regression analysis [15]. This finding from one previous Chinese study suggested that the relation of SUA levels with BP might be explained by the obvious correlation of BMI with both BP and SUA levels. However, the current study showed that BP was positively correlated with SUA levels. Moreover, an increase in BP was significantly associated with an increased odds of hyperuricemia.

In healthy young black and white adults aged 18–30 years from the Coronary Artery Risk Development in Young Adults study, 10-year changes in serum TG levels were strictly related to changes in SUA levels [16]. In the Japanese-American population in Hawaii and among Japanese male office workers in Osaka, a positive association was shown between SUA and TG levels [11, 17]. SUA levels were also found to be inversely correlated with HDL-c levels in adolescents and adults [18]. However, a previous investigation also highlighted that an unclear relationship existed between SUA levels and lipid metabolism [19]. Positive associations of SUA levels with TG and LDL-c levels as well as a negative association between SUA and HDL-c levels were significant in the Chinese cohorts in the current study. Moreover, we found that an increase in TG and LDL-c levels and a decrease in HDL-c levels were significantly associated with an increased odds of hyperuricemia.

In a survey of middle-aged men from British towns, SUA levels increased with increasing FBG levels up to approximately 8.0 mmol/L; at higher levels of FBG, SUA levels decreased [20]. Joint research from Sweden and United Kingdom showed an increase in SUA levels with increasing FBG levels up to 7.0 mmol/L in men and 9.0 mmol/L in women, and thereafter increasing FBG levels were accompanied by a significant decrease in SUA levels [21]. The current study confirmed negative associations of FBG levels with SUA levels and hyperuricemia.

Our study has a limitation. Because of the cross-sectional design, our results could not suggest any cause-effect mechanism linking the presence of increased SUA levels to cardiometabolic risk factors.

## Conclusions

This study shows that in a Chinese community-dwelling population, age, sex, BMI, BP, TG, HDL-c, LDL-c and FBG levels are cardiometabolic risk factors that are significantly associated not only with SUA levels, as well as with the presence of hyperuricemia.

## Abbreviations

SUA: Serum uric acid
BP: Blood pressure
BMI: Body mass index
FBG: Fasting blood glucose
TG: Triglyceride
HDL-c: High-density lipoprotein-cholesterol
LDL-c: Low-density lipoprotein-cholesterol

## References

[1] Chang HY, Pan WH, Yeh WT, Tsai KS (2001). Hyperuricemia and gout in Taiwan: results from the Nutritional and Health Survey in Taiwan (1993–96). J Rheumatol.

[2] Conen D, Wietlisbach V, Bovet P, Shamlaye C, Riesen W, Paccaud F (2004). Prevalence of hyperuricemia and relation of serum uric acid with cardiovascular risk factors in a developing country. BMC Public Health.

[3] Forman JP, Choi H, Curhan GC (2007). Plasma uric acid level and risk for incident hypertension among men. J Am Soc Nephrol.

[4] Chien KL, Chen MF, Hsu HC, Chang WT, Su TC, Lee YT (2008). Plasma uric acid and the risk of type 2 diabetes in a Chinese community. Clin Chem.

[5] Bickel C, Rupprecht HJ, Blankenberg S, Rippin G, Hafner G, Daunhauer A (2002). Serum uric acid as an independent predictor of mortality in patientswith angiographically proven coronary artery disease. Am J Cardiol.

[6] Li Y, Stamler J, Xiao Z, Folsom A, Tao S, Zhang H (1997). Serum uric acid and its correlates in Chinese adult populations, urban and rural, of Beijing. The PRC-USA Collaborative Study in Cardiovascular and Cardiopulmonary Epidemiology. Int J Epidemiol.

[7] Fang J, Alderman MH (2000). Serum uric acid and cardiovascular mortality the NHANES I epidermiologic follow-up study, 1971–1992. National Health and Nutrition Examination Survey. JAMA.

[8] Culleton B, Larson M, Kannel W, Levy D (1999). Serum uric acid and risk for cardiovascular diasease and death: the Framingham heart study. Ann Intern Med.

[9] Feig DI, Kang DH, Johnson RJ (2008). Uric acid and cardiovascular risk. N Engl J Med.

[10] Mikkelsen WM, Dodge HJ, Valkenburg H (1965). The distribution of serum uric acid values in a population unselected as to gout or hyperuricaemia, Tecumseh, Michigan 1959–1960. Am J Med.

[11] Yano K, Hoads G, Kagan A (1977). Epidemiology of serum urate levels among 8000 Japanese-American men in Hawaii. J Chronic Dis.

[12] Tuomilehto J, Zimmet P, Wolf E, Taylor R, Ram P, King H (1988). Plasma uric acid level and its association with diabetes mellitus and some biological parameters in a biracial population of Fiji. Am J Epidemiol.

[13] Selby JV, Friedman GD, Quesenberry CP (1990). Precursors of essential hypertension: pulmonary function, heart rate, uric acid, serum cholesterol, and other serum chemistries. Am J Epidemiol.

[14] Goldbourt U, Medalie JH, Herman JB, Neufeld HN (1980). Serum uric acid: correlation with biochemical, anthropometric, clinical and behavioral parameters in 10 000 Israeli men. J Chron Dis.

[15] Okada M, Takeshita M, Ueda K, Omae T, Hirota Y (1980). Factors influencing the serum uric acid level. A study based on a population survey in Hisayama town, Kyushu, Japan. J Chron Dis.

[16] Rathmann W, Funkhouser E, Dyer AR, Roseman JM (1998). Relations of hyperuricemia with the various components of the insulin resistance syndrome in young black and white adults: the CARDIA study. Ann Epidemiol.

[17] Nakanishi N, Suzuki K, Kawashimo H, Nakamura K, Tatara K (1999). Serum uric acid: correlation with biological, clinical and behavioral factors in Japanese men. J Epidemiol.

[18] Onat A, Uyarel H, Hergenc¸ G, Karabulut A, Albayrak S, Sari I (2006). Serum uric acid is a determinant ofmetabolic syndrome in a population-based study. Am J Hypertens.

[19] Lippi G, Montagnana M, Luca Salvagno G, Targher G, Cesare Guidi G (2010). Epidemiological association between uric acid concentration in plasma, lipoprotein(a), and the traditional lipid profile. Clin Cardiol.

[20] Cook DG, Shaper AG, Thelle DS, Whitehead TP (1986). Serum uric acid, serum glucose and diabetes: relationships in a population study. Postgrad Med J.

[21] Whitehead TP, Jungner I, Robinson D, Kolar W, Pearl A, Hale A (1992). Serum urate, serum glucose and diabetes. Ann Clin Biochem.

